# β-TrCP1 degradation is a novel action mechanism of PI3K/mTOR inhibitors in triple-negative breast cancer cells

**DOI:** 10.1038/emm.2014.127

**Published:** 2015-02-27

**Authors:** Yong Weon Yi, Hyo Jin Kang, Edward Jeong Bae, Seunghoon Oh, Yeon-Sun Seong, Insoo Bae

**Affiliations:** 1 Department of Oncology, Lombardi Comprehensive Cancer Center, Georgetown University Medical Center, Washington, DC, USA; 2 Department of Nanobiomedical Science & BK21 PLUS Research Center for Regenerative Medicine, Dankook University, Cheonan, Korea; 3 Department of Nursing and Health Studies, Georgetown University, Washington, DC, USA; 4 Department of Physiology, College of Medicine, Dankook University, Cheonan, Korea

## Abstract

An F-box protein, β-TrCP recognizes substrate proteins and destabilizes them through ubiquitin-dependent proteolysis. It regulates the stability of diverse proteins and functions as either a tumor suppressor or an oncogene. Although the regulation by β-TrCP has been widely studied, the regulation of β-TrCP itself is not well understood yet. In this study, we found that the level of β-TrCP1 is downregulated by various protein kinase inhibitors in triple-negative breast cancer (TNBC) cells. A PI3K/mTOR inhibitor PI-103 reduced the level of β-TrCP1 in a wide range of TNBC cells in a proteasome-dependent manner. Concomitantly, the levels of c-Myc and cyclin E were also downregulated by PI-103. PI-103 reduced the phosphorylation of β-TrCP1 prior to its degradation. In addition, knockdown of β-TrCP1 inhibited the proliferation of TNBC cells. We further identified that pharmacological inhibition of mTORC2 was sufficient to reduce the β-TrCP1 and c-Myc levels. These results suggest that mTORC2 regulates the stability of β-TrCP1 in TNBC cells and targeting β-TrCP1 is a potential approach to treat human TNBC.

## Introduction

Triple-negative breast cancers (TNBCs), which were first introduced in the medical literature in 2005, are a heterogeneous group of tumors that are immunohistologically defined as the lack of estrogen receptor (ER) and progesterone receptor (PR) expression, as well as human epidermal growth factor receptor 2 expression/amplification.^1^ Despite marked increase of studies on TNBCs during the past decade, our knowledge of how TNBCs can be treated is still limited.^2, 3, 4^ Approximately 15 to 20% of all breast cancers are diagnosed as TNBCs.^4^ A systemic review demonstrated the highest incidence of TNBCs in women of African ancestry (26.99%) followed by Hispanic (17.5%), Asian (12.19%), Caucasian (11.73%) and other women (8.42%).^5^ A recent meta-analysis of large data sets revealed that TNBCs are classified in at least six distinct molecular subtypes that include two basal-like, an immunomodulatory, a mesenchymal, a mesenchymal stem-like and a luminal androgen receptor subtype.^6^ However, no successful therapeutic target is currently available to treat TNBC patients.^2, 3, 4^

Beta-transducin repeat containing proteins (β-TrCPs) are members of the F-box/WD repeat-containing protein (FBXW) subfamily of F-box protein families.^7, 8, 9^ As an F-box protein, the β-TrCP is the substrate-recognition subunit of SKP1-cullin 1-F-box protein, E3 ligase complexes and well conserved across species.^8, 9^ In humans, β-TrCP exists as two homologues, β-TrCP1 (also known as FBXW1) and β-TrCP2 (also known as FBXW11), which are encoded by two distinct genes but share extensive amino acid sequence homology. The differences between these two proteins still remain elusive.^8, 9^ The function of β-TrCPs in tumorigenesis is either oncogenic or tumor-suppressive in a tissue-specific or cellular context-dependent manner homology.^8, 9^ Although it has been widely studied that β-TrCP recognizes diverse proteins and regulates their stability, the regulation of β-TrCP itself is not yet understood.

Here, we demonstrated that the expression of β-TrCP1 protein is regulated by mTORC2 and targeting β-TrCP1 is a potential therapeutic approach to treat TNBC cells.

## Materials and methods

### Cell culture and reagents

Cell culture reagents were purchased from Invitrogen (Carlsbad, CA, USA), Lonza (Basel, Switzerland), or Cellgro (Manassas, VA, USA). All cells, except for SUM149PT, were obtained from the Tissue Culture Shared Resource of Georgetown University Medical Center (Washington, DC, USA) and maintained in the Dulbecco's Modified Eagle Medium (DMEM; Lonza) containing 10% heat inactivated fetal bovine serum (FBS; HyClone, Logan, UT, USA or Omega Scientific, Tarzana, CA, USA) and 100 units ml^−1^ penicillin/streptomycin (Lonza). SUM149PT was maintained according to manufacturer's recommendation (Asterand, Detroit, MI, USA). The viability of cultured cells was monitored by the trypan blue dye-exclusion method using the Luna Automated Cell Counter (Logos Biosystems, Gyeonggi-do, Korea). Protein kinase inhibitors were purchased from the following sources: CHIR-99021, GDC-0941, GSK1059615, IC-87114, MK-2206, PI-103, PIK-75, PIK-90, TG100-115, TGX221 and WYE-354 from Selleck Chemicals (Houston, TX, USA); BEZ235 and ZSTK474 from LC Labs (Woburn, MA, USA). Bafilomycin A1, MG132 and rapamycin were purchased from Sigma (St Louis, MO, USA). Stock solutions of compounds, except for BEZ235, were made in dimethyl sulfoxide and stored at −20 °C in small aliquots. BEZ235 was dissolved in dimethylformamide.

### MTT (3-(4,5-Dimethylthiazol-2-yl)-2,5-diphenyltetrazolium bromide) cell viability assays

Cell viability was determined at ~72 h after treatment of compounds by MTT assay as described previously.^10, 11^ The EC_50_ values were calculated by CompuSyn software V1.0 (ComboSyn, Paramus, NJ, USA).

### Western blots and antibodies

Western blot analyses were performed as described previously.^10^ Antibodies used in this study were as follows: phospho-AKT (Ser473; #9271), AKT (#9272), phospho-GSK3β (S9; #9323), LC3B (#3868), phospho-mTOR (S2448; #2971), mTOR (#4517), p-S6 (S235/S236; #4856), S6 (#2217) and β-TrCP (#4394) from Cell Signaling (Danvers, MA, USA); β-TrCP (sc-390629), HSP90 (sc-7947), c-Myc (sc-764) from Santa Cruz (Santa Cruz, CA, USA); cyclin E (51-1459GR) from BD Biosciences (San Jose, CA, USA) and β-actin and horseradish peroxidase-conjugated secondary antibodies from Sigma. Densitometric analysis was performed by ImageJ (NIH, Bethesda, MD, USA).^12^

### Immunoprecipitation

Immunoprecipitation was performed as described previously^13^ with phospho-(Ser/Thr) Phe (#9631) antibody. Then, immune complexes were dissolved on SDS-poly acrylamide gel electrophoresis and western blot analysis was performed with mouse β-TrCP antibody (sc-390629) from Santa Cruz.

### Transfection of small interference RNA and cell proliferation assay

Transfection of small interference RNA (siRNA) was performed with Lipofectamine 2000 (Invitrogen) as described previously.^14^ In brief, HS578T (0.4 × 10^5^ cells per well) or MDA-MB-231 (1.0 × 10^5^ cells per well) cells in six-well plates were transfected with 100 pmoles of siRNA and 2.5 μl of Lipofectamine 2000 in serum-free DMEM. After 4 h incubation, cells were supplemented with equal volume of DMEM containing 20% FBS and 200 units ml^−1^ penicillin/streptomycin to maintain final 10% FBS and further incubated for 3 days. After 3-day incubation, cells were further supplemented with equal volume of DMEM containing 20% FBS and 200 units ml^−1^ penicillin/streptomycin and incubated up to two more days. The cells were trypsinized at indicated days and the number of viable cells was determined by counting cells which were stained by acridine orange/propidium iodide with the Luna-FL Dual Fluorescence Cell Counter (Logos Biosystems). The siRNAs were purchased from Bioneer (Seoul, Korea) with following sequences: control-siRNA 5′-GAC GAG CGG CAC GUG CAC AUU-3′ β-TrCP1-siRNA #3, 5′-CUC AGA GAG AGA AGA CUG U(dTdT)-3′ β-TrCP1-siRNA #4, 5′-GUG AUG UGU AGU CAG UGU A(dTdT)-3′ β-TrCP2-siRNA #3, 5′-GAA UUC UGA CUU GUU GUA U(dTdT)-3′ and β-TrCP2-siRNA #9, 5′-CAG ACA ACC UUU GAA UUG U(dTdT)-3′.

## Results

### A PI3K/mTOR inhibitor PI-103 reduces the viable cells of TNBC cell lines

**Figure 1:**
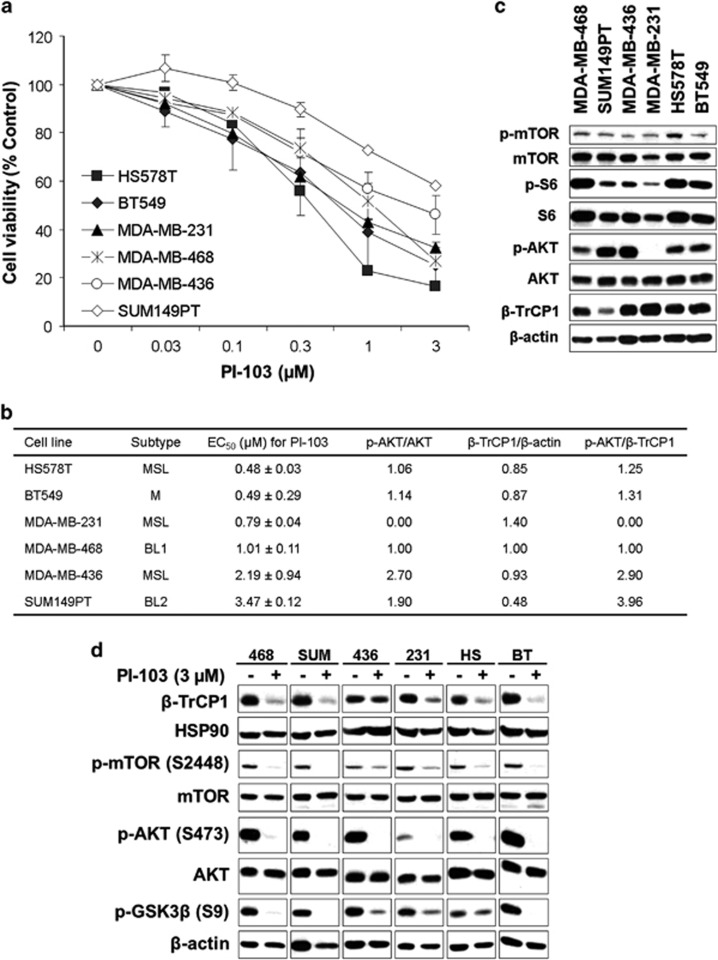
PI-103 downregulates the level of β-TrCP1 protein in TNBC cells. (**a**) PI-103 reduced the viable TNBC cells in a dose-dependent manner. Cells were treated with increasing amounts of PI-103 up to 72 h and the viable cells were measured by MTT assay. Data from two independent experiments performed in triplicate are shown as mean±s.e.m. (**b**) EC_50_ values of PI-103 and the relative protein expression in TNBC cells. EC_50_ values were obtained from data in **a** and the relative protein expression was obtained by densitometric analysis of data in **c**. (**c**) The levels of protein expression in TNBC cells. Cell lysates from exponentially growing cells were subjected to western blot analysis with indicated antibodies. β-actin was used as a loading control. (**d**) PI-103 reduced the level of β-TrCP1 protein in TNBC cells. Cells were treated with 3 μM of PI-103 for 24 h and subjected to western blot analysis with indicated antibodies. HSP90 or β-actin was used as a loading control.

During our previous study,^10^ we noticed that a PI3K/mTOR inhibitor PI-103^15^ reduced the cell viability of TNBC cells in a dose-dependent manner. As TNBC is a heterogeneous disease which can be further subgrouped into six subtypes,^4^ we further tested the effect of PI-103 with expanded TNBC cell lines by MTT assay. As shown in Figure 1a, PI-103 significantly reduced the number of viable cells in a dose-dependent manner in all of the cell lines tested. There is no significant correlation between the EC_50_ values for PI-103 and the subtypes of these cell lines (Figure 1b). To further compare the level of proteins in the PI3K/mTOR pathway in these cells, we performed western blot analysis (Figure 1c). All these cells expressed detectable levels of AKT downstream proteins such as phospho-mTOR (Y2448) and p-S6 (S235/S236). As reported previously,^16^ BRCA1-defective cells exhibited higher level of phospho-AKT (S473) than other TNBC cells. On the contrary, the expression of phospho-AKT (S473) was barely detectable in MDA-MB-231 cells. Interestingly, significant level of β-TrCP1 protein was detected in all these cells. There is no significant correlation between the EC_50_ values of PI-103 and the expression levels of these proteins, whereas high ratio of phospho-AKT to β-TrCP1 correlated with high EC_50_ values for PI-103 (Figure 1b).

To determine the effect of PI-103 on the level of these proteins, we performed western blot analysis with cell lysates from cells treated with 3 μM of PI-103 for 24 h (Figure 1d). As expected, PI-103 reduced the phosphorylation level of AKT substrates including mTOR (S2448)^17^ and GSK3β (S9)^18^ in all cell lines test. PI-103 also reduced the level of mTORC2 substrate, phospho-AKT (S473).^19^ Unexpectedly, the level of β-TrCP1 was also reduced by PI-103 in these cells (Figure 1d).

### PI-103 reduces the level of β-TrCP, c-Myc and cyclin E in TNBC cells

**Figure 2:**
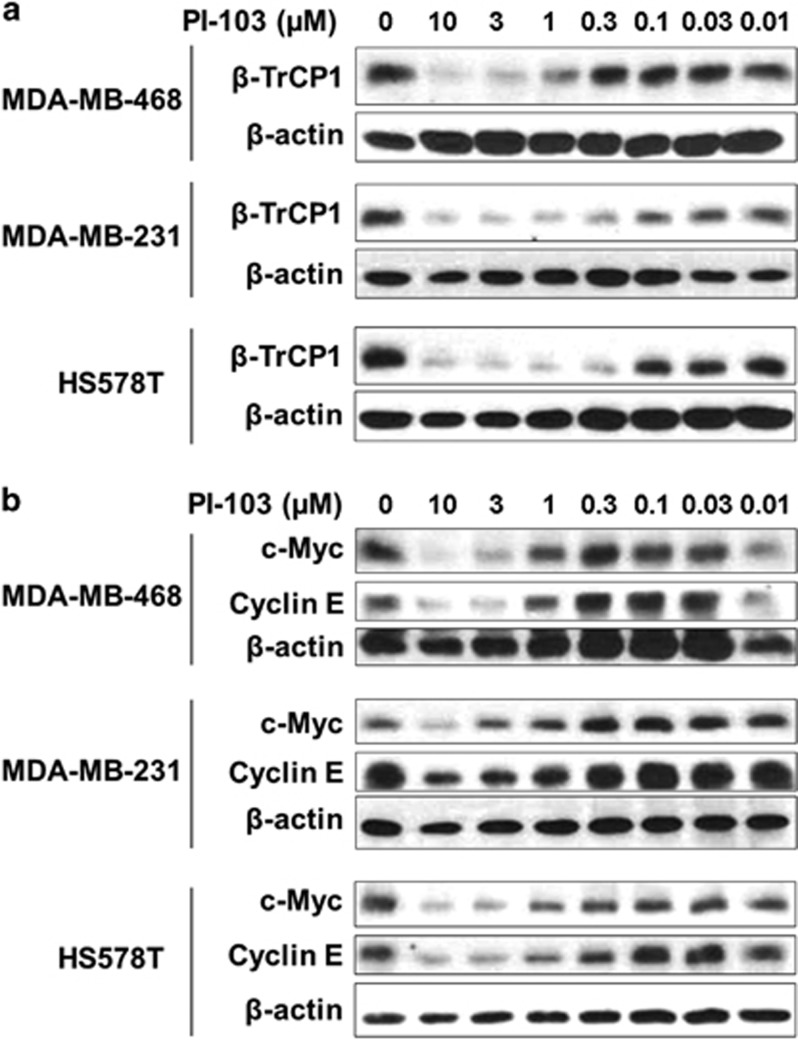
PI-103 downregulates the levels of β-TrCP1, c-Myc and cyclin E proteins in TNBC cells. (**a**, **b**) Cells were treated with increasing concentrations of PI-103 for 24 h and the cell lysates were subjected to western blot analysis with indicated antibodies. β-actin was used as a loading control.

The effect of PI-103 on the level of β-TrCP1 was further determined by western blot analyses. Three TNBC cell lines were treated with increasing concentrations of PI-103 for 24 h and western blot analyses were performed. Consistently, the level of β-TrCP1 was reduced by PI-103 in a dose-dependent manner in all these cells (Figure 2a). It has been reported that β-TrCP stabilizes c-Myc through antagonizing FBW7-mediated turnover.^20^ It has been also demonstrated that activation of Ras/PI3K/ERK pathway induces c-Myc stabilization in melanoma cells,^21^ and inhibition of PI3K/AKT pathway by LY294002 in melanoma cells,^21^ or by PI-103 in Burkitt's lymphoma cells,^22^ and reduces c-Myc expression. Consistently, the levels of c-Myc and its downstream target, cyclin E, were also reduced by PI-103 in a dose-dependent manner (Figure 2b).

**Figure 3:**
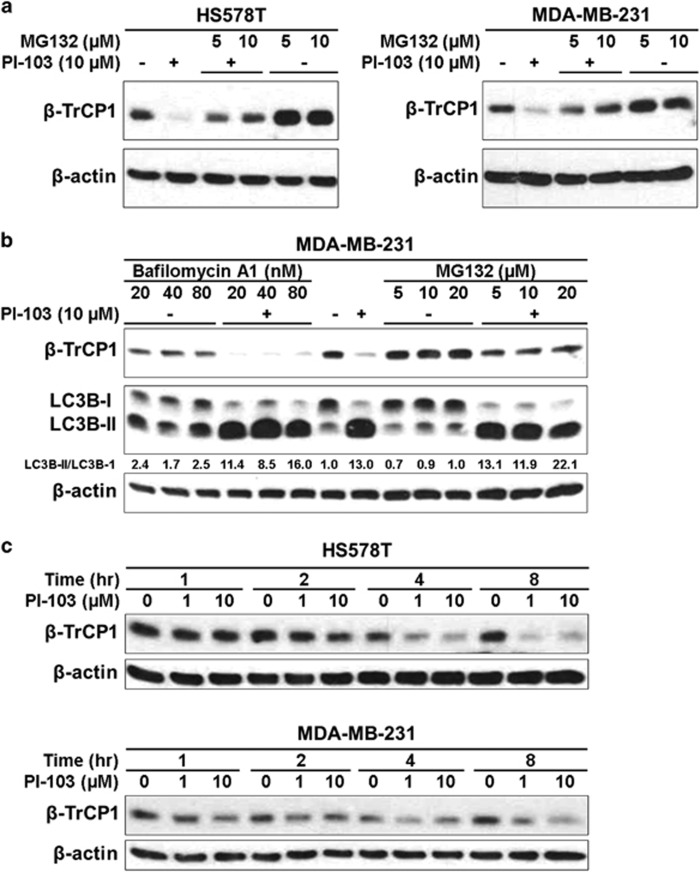
PI-103 downregulates the level of β-TrCP1 protein via proteasome-dependent manner. (**a**) Cells were treated with 10 μM of PI-103 for 20 h and further incubated for 4 h in the presence of different concentrations of MG-132. (**b**) Cells were treated with 10 μM of PI-103 for 20 h and further incubated for 4 h in the presence of different concentrations of bafilomycin A1 or MG-132. (**c**) Cells were treated with PI-103 for indicated time. (**a**–**c**) Western blot analysis was performed with indicated antibodies. β-actin was used as a loading control.

To determine whether the reduction of β-TrCP1 is dependent on proteasome-mediated degradation, western blot analysis was conducted as follows: HS578T and MDA-MB-231 cells were treated with PI-103 for 24 h. To inhibit proteasome-dependent proteolysis, MG132 was added to the cells 4 h before harvest. Western blot analysis revealed that PI-103-mediated reduction of β-TrCP1 was reversed by MG132 in these cells (Figure 3a). In addition, MG132 treatment increased the basal level of β-TrCP1 in the absence of PI-103 (Figure 3a).

Although it has been reported that PI-103 induces autophagy in glioma cells,^17^ we further accessed the effect of bafilomycin A1, an inhibitor of autophagic vacuole maturation^23^ on the degradation of β-TrCP1 by PI-103. MDA-MB-231 cells were treated with PI-103 for 24 h and either bafilomycin A1 or MG132 was treated for 4 h before harvest. As expected, bafilomycin A1 increased the accumulation of LC3B-II, a marker of autophagy, in the absence of PI-103 (Figure 3b). However, bafilomycin A1 did not affect the PI-103-mediated degradation of β-TrCP1. On the contrary, MG132 reversed the effect of PI-103 on the level of β-TrCP1 (Figure 3b, lanes 8 and 12–14) without affecting the accumulation of LC3B-II in the absence of PI-103 (Figure 3b, lanes 7 and 9–11). In these conditions, the level of β-TrCP1 was increased by MG132 even in the absence of PI-103. These implicate the basal level of β-TrCP1 was regulated by MG132 in these cells. All these results suggest that PI-103 reduced the level of β-TrCP1 by a proteasome-mediated degradation. The degradation of β-TrCP1 was observed as early as 4 h after PI-103 treatment (Figure 3c). Although we cannot completely exclude the possibility of mRNA reduction by PI-103 treatment, rapid reduction of the level of β-TrCP1 in the time-course treatment of β-TrCP1 (Figure 3c) suggests that proteasomal degradation is a major mechanism of β-TrCP1 reduction by PI-103.

### PI-103 reduces the phosphorylation of β-TrCP1

**Figure 4:**
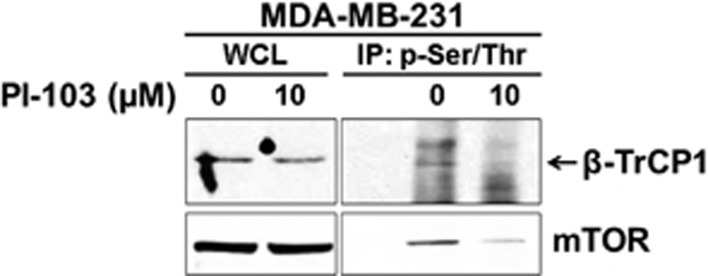
PI-103 reduces the phosphorylation of β-TrCP1. MDA-MB-231 cells were treated with PI-103 for 2 h and immunoprecipitation of phospho-proteins was performed as described in material and methods. Western blot analysis was performed with indicated antibodies.

The β-TrCP protein has several potential phosphorylation sites (data not shown). To determine whether β-TrCP1 is phosphorylated or not, we performed western blot analysis after immunoprecipitation of phospho proteins. To avoid β-TrCP1 degradation, MDA-MB-231 cells were treated with 10 μM of PI-103 for 2 h and phospho-proteins were immuneprecipitated by phospho-Ser/Thr Phe antibody. Then the immune complexes were analyzed by western blot with β-TrCP1 antibody. As shown in Figure 4, β-TrCP1 was phosphorylated in MDA-MB-231 cells and brief treatment of PI-103 reduced this phosphorylation. As a control, the level of mTOR protein was also determined. As expected the phospho-mTOR (S2448) was reduced by PI-103 treatment without affecting the level of mTOR.

### Knockdown of β-TrCP1 reduces the proliferation of TNBC cells

**Figure 5:**
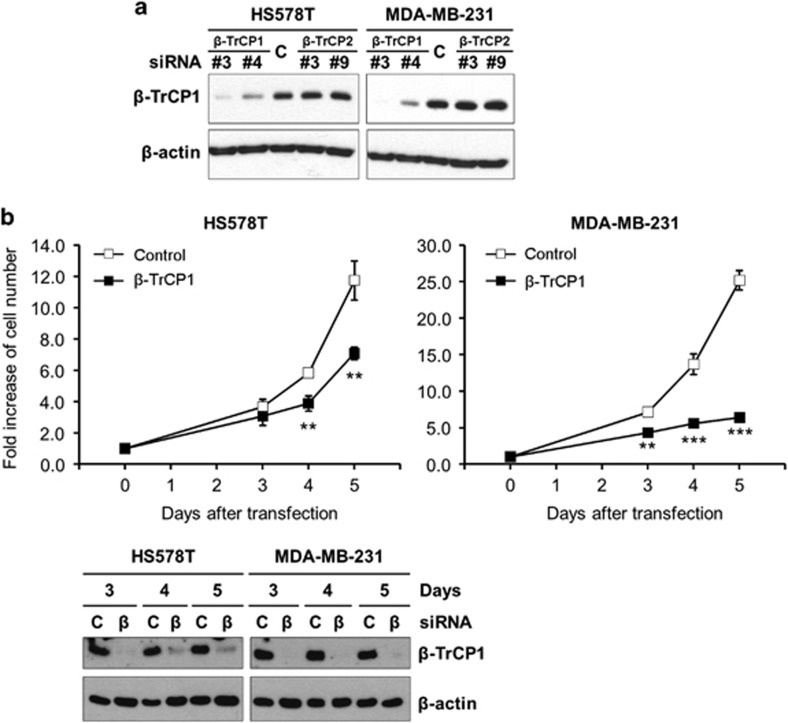
Knockdown of β-TrCP1 inhibits the proliferation of TNBC cells. (**a**) HS578T and MDA-MB-231 cells were transfected with siRNAs as indicated for 3 days and western blot analysis was performed with indicated antibodies. β-actin was used as a loading control. (**b**) HS578T and MDA-MB-231 cells were transfected with β-TrCP1-siRNA #3 as described in Materials and methods and the number of viable cells was determined by acridine orange/propidium iodide staining. Data from two independent experiments performed in triplicate are shown as mean±s.e.m. **P<0.01 and ***P<0.001. Western blot analysis of transfected cells was performed with indicated antibodies. β-actin was used as a loading control.

As PI-103 reduced the viable cell numbers and the level of β-TrCP1 in a broad range of TNBC cells, we questioned whether β-TrCP1 has a role in the proliferation of TNBC cells. To address this question, we performed knockdown of β-TrCP1 by siRNA and determined its effect on the proliferation of TNBC cells. HS578T and MDA-MB-231 cells were transfected with specific siRNAs for β-TrCP1 and for β-TrCP2 as a control and western blot analysis was performed. As shown in Figure 5a, β-TrCP1-siRNAs reduced the level of β-TrCP1.

Next, we determined the number of viable cells at various time points after β-TrCP1 knockdown in HS578T and MDA-MB-231 cells. Interestingly, knockdown of β-TrCP1 was enough to reduce the proliferation of these cells (Figure 5b). The anti-proliferative effect was evident at 3 or 4 days after β-TrCP1 knockdown and sustained up to 5 days. Western blot analysis revealed that the level of β-TrCP1 was slightly reversed over the time in HS578T cells, whereas no significant restoration of β-TrCP1 was observed in MDA-MB-231 cells. Consistent with these, the anti-proliferative effect of β-TrCP1 knockdown was more profound in MDA-MB-231 cells.

### Pharmacological inhibition of mTORC2 reduces the level of β-TrCP1 protein

**Figure 6:**
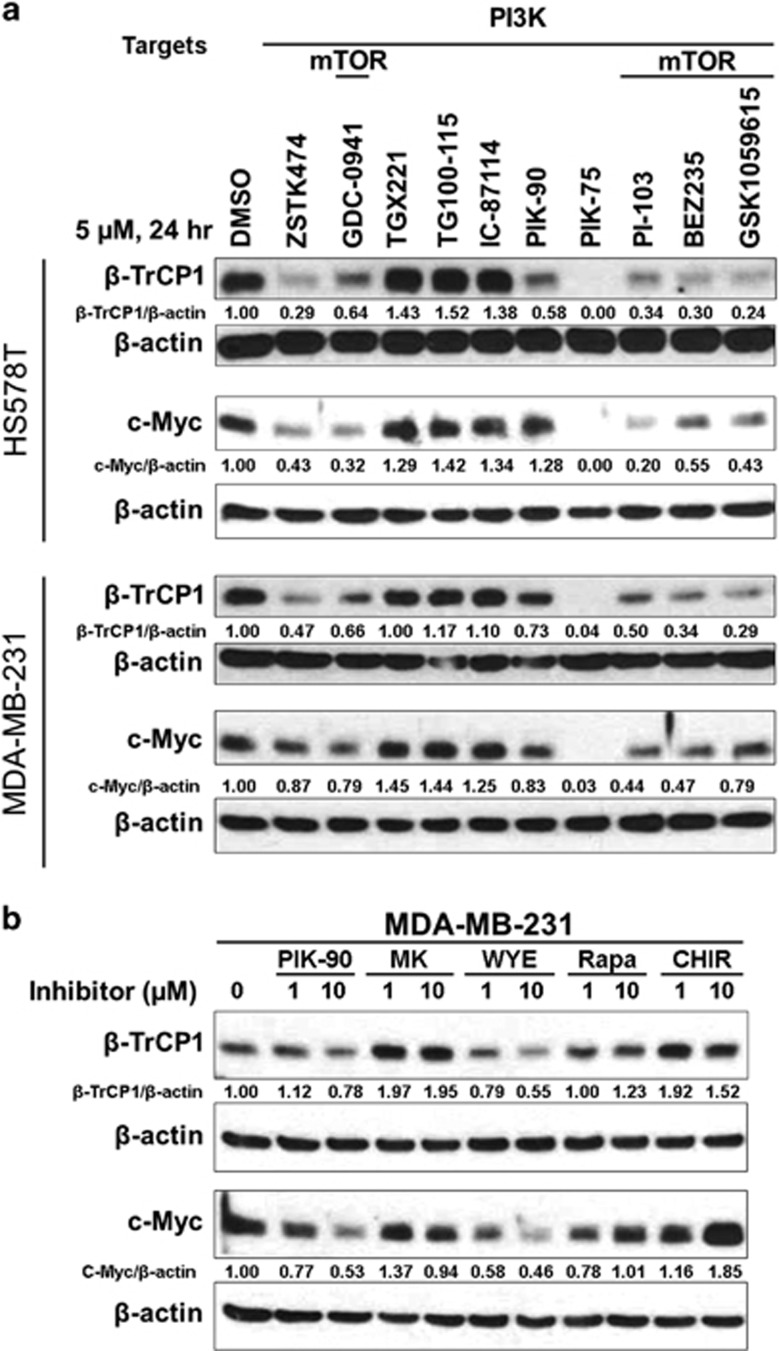
PI3K/mTOR inhibitors downregulate the levels of β-TrCP1 and c-Myc proteins in TNBC cells. (**a**) Cells were treated with 5 μM of compounds for 24 h and the protein expression was determined by western blot analysis with indicated antibodies. (**b**) Cells were treated with 1 or 10 μM of compounds for 24 h and the protein expression was determined by western blot analysis with indicated antibodies. (**a**, **b**) β-actin was used as a loading control.

To identify the kinase responsible for the phosphorylation of β-TrCP1, HS578T and MDA-MB-231 cells were treated with various PI3K/mTOR inhibitors for 24 h. As shown in Figure 6a, most of the PI3K inhibitors reduced the level of β-TrCP1 in these cells. Notably, PI3K inhibitors, which are known to inhibit preferentially PI3Kα, markedly reduced the β-TrCP1 protein, whereas PI3K inhibitors (TGX221, TG100-115 and IC-87114) specific to other isoforms (β, δ and γ)^15, 24^ exhibited little or no effect on the level of β-TrCP1 protein. More interestingly, all of the PI3K/mTOR dual inhibitors, such as GDC-0941,^25^ PI-103,^15^ BEZ235^26^ and GSK1059615^27^ invariantly reduced the level of β-TrCP1 protein (Figure 6a). Again, the level of c-Myc was concomitantly reduced by inhibitors which reduced the level of β-TrCP1.

To further dissect the pathway to β-TrCP1 degradation, MDA-MB-231 cells were treated with different concentrations of more specific inhibitors. As shown in Figure 6b, a PI3K inhibitor PIK-90 and mTOR-kinase inhibitor WYE-354 ^28^ reduced the levels of β-TrCP1 and c-Myc in a dose-dependent manner. On the contrary, the level of β-TrCP1 protein was rather induced by other inhibitors including an allosteric AKT inhibitor MK-2206,^29^ an allosteric mTORC1 inhibitor rapamycin,^30^ and a GSK3α/β inhibitor CHIR-99021.^31^ As an mTOR kinase inhibitor, WYE-354 inhibits both mTORC1 and mTORC2,^28^ whereas rapamycin more specifically inhibits mTORC1.^30^ Taken together, we speculated that mTORC2 is a potential kinase that stabilizes the β-TrCP1 protein in TNBC cells.

## Discussion

In the present study, we identified β-TrCP1 as a potential target to treat the TNBC cells. Inhibition of PI3K/mTOR by PI-103 reduced the level of β-TrCP1 in diverse TNBC cell lines in a proteasome-dependent manner. siRNA-based depletion of β-TrCP1 profoundly reduced the proliferation of TNBC cell lines, HS578T and MDA-MB-231. Pharmacological inhibition of mTORC2 but not mTORC1 was sufficient to reduce the level of β-TrCP1 and c-Myc in TNBC cells.

**Figure 7:**
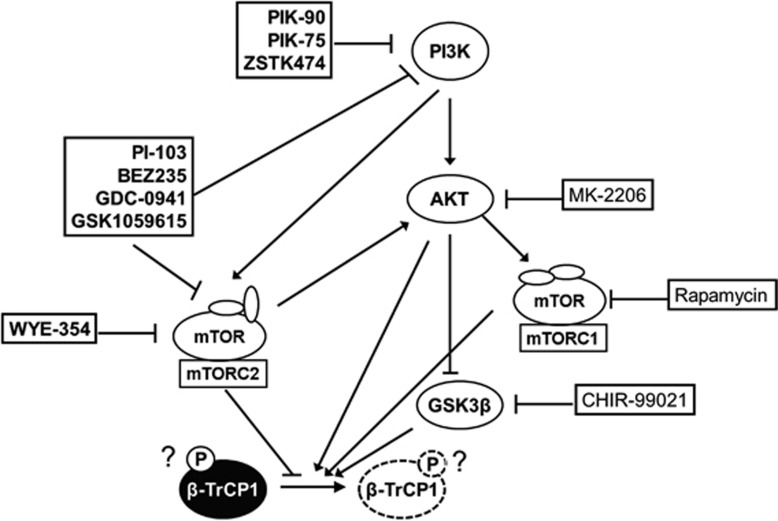
Schematic diagram for proposed regulation of β-TrCP1 by upstream kinases.

Treatment of a series of specific kinase inhibitors revealed that mTORC2 is a plausible regulator of β-TrCP1 stability in TNBC cells. Treatment of PI-103 reduced the level of phospho-β-TrCP1. PI3K/mTOR inhibitors reduced the level of β-TrCP1, whereas MK-2206 (an allosteric AKT inhibitor) did not reduce the β-TrCP1 protein in TNBC cells. Because the inhibition of PI3K reduces the activity of AKT that activates mTORC1 activity, these results are somewhat complicated. However, a recent report suggested that the activity of mTORC2 can be directly regulated by PI3K in a TSC-independent manner.^32, 33^ In addition, WYE-354, an mTOR-kinase inhibitor, reduced the level of β-TrCP1. On the contrary, rapamycin, an mTORC1 inhibitor, did not affect the β-TrCP1 level. All these results suggest that the level of β-TrCP1 protein is regulated by mTORC2 in TNBC cells (Figure 7). Future studies with specific siRNAs for components of PI3K/mTORC2 pathway will further delineate the signaling pathway that is important to regulate β-TrCP1 stability.

The stability of F-box itself is also regulated by post-translational regulation. As an example, S-phase kinase-associated protein 2, another oncogenic F-box protein, has been reported to be phosphorylated and stabilized by AKT1 in human cancers.^34^ However, the regulation of β-TrCP1 stability through post-translational modification has not been attributed yet. Our present data, which demonstrates that PI-103 reduced the level of phospho-β-TrCP1, suggest that the stability of β-TrCP1 might be also regulated by protein modification. Recently, S-phase kinase-associated protein 2 has been identified as an E3 ligase that is responsible for degradation of β-TrCP.^35^ In fact, β-TrCP itself is an E3 ligase for degradation of Emi1,^36, 37^ an inhibitor of the APC/C E3 ligase which mediates S-phase kinase-associated protein 2 degradation.^38, 39^ More recently, SIRT1 has been identified as a negative regulator of β-TrCP through protein degradation.^40^

Although reported to function as a tissue-specific tumor suppressor, multiple studies have supported that β-TrCP has roles as an oncogene and promotes tumorigenesis when overexpressed and elevated levels of β-TrCP has been reported various human cancers including hepatoblastoma, melanoma, colorectal, pancreatic, prostate, breast and gastric cancers.^8, 9^ In fact, potential tumor-suppressor function is a common property of many β-TrCP substrates such as inhibitor of nuclear factor-κB (IκB), programmed cell-death protein 4 (PDCD4), DEP domain-containing mTOR-interacting protein (DEPTOR), RE1-silencing transcription factor (REST) and metastasis suppressor 1 (MTSS1).^8, 9, 41, 42^ Interestingly, phosphorylation of DEPTOR, a negative regulator of mTORC1/2, generates phosphodegron that binds β-TrCP and leads to degradation of DEPTOR.^43, 44, 45^ In addition, depletion of β-TrCP by shRNA induced accumulation of DEPTOR, reduced mTOR and S6 kinase activity and activated autophagy to reduce cell growth.^44, 45^ These results suggest that β-TrCP1 is an important candidate of druggable target protein to treat cancers via modulating cell proliferation and/or survival.

## References

[bib1] Brenton JD Carey LA Ahmed AA Caldas C Molecular classification and molecular forecasting of breast cancer: ready for clinical application J Clin Oncol 2005 23 7350 7360 16145060 10.1200/JCO.2005.03.3845

[bib2] Clark O Botrel TE Paladini L Ferreira MB Targeted therapy in triple-negative metastatic breast cancer: a systematic review and meta-analysis Core Evid 2014 9 1 11 24476748 10.2147/CE.S52197 PMC3891489

[bib3] Herold CI Anders CK New targets for triple-negative breast cancer Oncology 2013 27 846 854 24282978

[bib4] Lehmann BD Pietenpol JA Identification and use of biomarkers in treatment strategies for triple-negative breast cancer subtypes J Pathol 2014 232 142 150 24114677 10.1002/path.4280 PMC4090031

[bib5] Limbada R Slater A Jain A PB.53: are ethnic minorities more likely to develop triple-negative breast cancer? A systematic review Breast Cancer Res 2013 15 (Suppl 1 P53

[bib6] Lehmann BD Bauer JA Chen X Sanders ME Chakravarthy AB Shyr Y Identification of human triple-negative breast cancer subtypes and preclinical models for selection of targeted therapies J Clin Invest 2011 121 2750 2767 21633166 10.1172/JCI45014 PMC3127435

[bib7] Kipreos ET Pagano M The F-box protein family Genome Biol 2000 1 Review S3002 10.1186/gb-2000-1-5-reviews3002 PMC138887 11178263

[bib8] Wang Z Liu P Inuzuka H Wei W Roles of F-box proteins in cancer Nat Rev Cancer 2014 14 233 247 24658274 10.1038/nrc3700 PMC4306233

[bib9] Lau A Liu Y Tron A Inuzuka H Wei W The role of FBXW subfamily of F-box proteins in tumorigenesis. SCF and APC E3 ubiquitin ligases in tumorigenesis SpringerBriefs in Cancer Research Springer International Publishing: Berlin, Germany 2014 15 45

[bib10] Yi YW Hong W Kang HJ Kim HJ Zhao W Wang A Inhibition of the PI3K/AKT pathway potentiates cytotoxicity of EGFR kinase inhibitors in triple-negative breast cancer cells J Cell Mol Med 2013 17 648 656 23601074 10.1111/jcmm.12046 PMC3822817

[bib11] Yi YW Kang HJ Kim HJ Kong Y Brown ML Bae I Targeting mutant p53 by a SIRT1 activator YK-3-237 inhibits the proliferation of triple-negative breast cancer cells Oncotarget 2013 4 984 994 23846322 10.18632/oncotarget.1070 PMC3759676

[bib12] Schneider CA Rasband WS Eliceiri KW NIH Image to ImageJ: 25 years of image analysis Nat Methods 2012 9 671 675 22930834 10.1038/nmeth.2089 PMC5554542

[bib13] Kang HJ Hong YB Kim HJ Bae I CR6-interacting factor 1 (CRIF1) regulates NF-E2-related factor 2 (NRF2) protein stability by proteasome-mediated degradation J Biol Chem 2010 285 21258 21268 20427290 10.1074/jbc.M109.084590 PMC2898415

[bib14] Hou S Yi YW Kang HJ Zhang L Kim HJ Kong Y Novel carbazole inhibits phospho-STAT3 through induction of protein-tyrosine phosphatase PTPN6 J Med Chem 2014 57 6342 6353 24978112 10.1021/jm4018042 PMC5852381

[bib15] Knight ZA Gonzalez B Feldman ME Zunder ER Goldenberg DD Williams O A pharmacological map of the PI3-K family defines a role for p110alpha in insulin signaling Cell 2006 125 733 747 16647110 10.1016/j.cell.2006.03.035 PMC2946820

[bib16] Yi YW Kang HJ Kim HJ Hwang JS Wang A Bae I Inhibition of constitutively activated phosphoinositide 3-kinase/AKT pathway enhances antitumor activity of chemotherapeutic agents in breast cancer susceptibility gene 1-defective breast cancer cells Mol Carcinog 2013 52 667 675 22488590 10.1002/mc.21905 PMC3586771

[bib17] Sekulic A Hudson CC Homme JL Yin P Otterness DM Karnitz LM A direct linkage between the phosphoinositide 3-kinase-AKT signaling pathway and the mammalian target of rapamycin in mitogen-stimulated and transformed cells Cancer Res 2000 60 3504 3513 10910062

[bib18] Cross DA Alessi DR Cohen P Andjelkovich M Hemmings BA Inhibition of glycogen synthase kinase-3 by insulin mediated by protein kinase B Nature 1995 378 785 789 8524413 10.1038/378785a0

[bib19] Sarbassov DD Guertin DA Ali SM Sabatini DM Phosphorylation and regulation of Akt/PKB by the rictor-mTOR complex Science 2005 307 1098 1101 15718470 10.1126/science.1106148

[bib20] Popov N Schulein C Jaenicke LA Eilers M Ubiquitylation of the amino terminus of Myc by SCF(beta-TrCP) antagonizes SCF(Fbw7)-mediated turnover Nat Cell Biol 2010 12 973 981 20852628 10.1038/ncb2104

[bib21] Tsai WB Aiba I Long Y Lin HK Feun L Savaraj N Activation of Ras/PI3K/ERK pathway induces c-Myc stabilization to upregulate argininosuccinate synthetase, leading to arginine deiminase resistance in melanoma cells Cancer Res 2012 72 2622 2633 22461507 10.1158/0008-5472.CAN-11-3605 PMC3433038

[bib22] Spender LC Inman GJ Phosphoinositide 3-kinase/AKT/mTORC1/2 signaling determines sensitivity of Burkitt's lymphoma cells to BH3 mimetics Mol Cancer Res 2012 10 347 359 22241218 10.1158/1541-7786.MCR-11-0394 PMC3378513

[bib23] Yamamoto A Tagawa Y Yoshimori T Moriyama Y Masaki R Tashiro Y Bafilomycin A1 prevents maturation of autophagic vacuoles by inhibiting fusion between autophagosomes and lysosomes in rat hepatoma cell line, H-4-II-E cells Cell Struct Funct 1998 23 33 42 9639028 10.1247/csf.23.33

[bib24] Marone R Cmiljanovic V Giese B Wymann MP Targeting phosphoinositide 3-kinase: moving towards therapy Biochim Biophys Acta 2008 1784 159 185 17997386 10.1016/j.bbapap.2007.10.003

[bib25] Folkes AJ Ahmadi K Alderton WK Alix S Baker SJ Box G The identification of 2-(1H-indazol-4-yl)-6-(4-methanesulfonyl-piperazin-1-ylmethyl)-4-morpholin-4-yl-t hieno[3,2-d]pyrimidine (GDC-0941) as a potent, selective, orally bioavailable inhibitor of class I PI3 kinase for the treatment of cancer J Med Chem 2008 51 5522 5532 18754654 10.1021/jm800295d

[bib26] Maira SM Stauffer F Brueggen J Furet P Schnell C Fritsch C Identification and characterization of NVP-BEZ235, a new orally available dual phosphatidylinositol 3-kinase/mammalian target of rapamycin inhibitor with potent *in vivo* antitumor activity Mol Cancer Ther 2008 7 1851 1863 18606717 10.1158/1535-7163.MCT-08-0017

[bib27] Carnero A Novel inhibitors of the PI3K family Expert Opin Investig Drugs 2009 18 1265 1277 10.1517/13543780903066798 19589091

[bib28] Yu K Toral-Barza L Shi C Zhang WG Lucas J Shor B Biochemical, cellular, and *in vivo* activity of novel ATP-competitive and selective inhibitors of the mammalian target of rapamycin Cancer Res 2009 69 6232 6240 19584280 10.1158/0008-5472.CAN-09-0299

[bib29] Hirai H Sootome H Nakatsuru Y Miyama K Taguchi S Tsujioka K MK-2206, an allosteric Akt inhibitor, enhances antitumor efficacy by standard chemotherapeutic agents or molecular targeted drugs *in vitro* and *in vivo* Mol Cancer Ther 2010 9 1956 1967 20571069 10.1158/1535-7163.MCT-09-1012

[bib30] Nyfeler B Bergman P Triantafellow E Wilson CJ Zhu Y Radetich B Relieving autophagy and 4EBP1 from rapamycin resistance Mol Cell Biol 2011 31 2867 2876 21576371 10.1128/MCB.05430-11 PMC3133392

[bib31] Ring DB Johnson KW Henriksen EJ Nuss JM Goff D Kinnick TR Selective glycogen synthase kinase 3 inhibitors potentiate insulin activation of glucose transport and utilization *in vitro* and *in vivo* Diabetes 2003 52 588 595 12606497 10.2337/diabetes.52.3.588

[bib32] Dalle Pezze P Sonntag AG Thien A Prentzell MT Godel M Fischer S A dynamic network model of mTOR signaling reveals TSC-independent mTORC2 regulation Sci Signal 2012 5 ra25 22457331 10.1126/scisignal.2002469

[bib33] Wahane SD Hellbach N Prentzell MT Weise SC Vezzali R Kreutz C PI3K-p110-alpha-subtype signalling mediates survival, proliferation and neurogenesis of cortical progenitor cells via activation of mTORC2 J Neurochem 2014 130 255 267 24645666 10.1111/jnc.12718

[bib34] Gao D Inuzuka H Tseng A Wei W Akt finds its new path to regulate cell cycle through modulating Skp2 activity and its destruction by APC/Cdh1 Cell Div 2009 4 11 19549334 10.1186/1747-1028-4-11 PMC2708142

[bib35] Wei S Chu PC Chuang HC Hung WC Kulp SK Chen CS Targeting the oncogenic E3 ligase Skp2 in prostate and breast cancer cells with a novel energy restriction-mimetic agent PLoS ONE 2012 7 e47298 23071779 10.1371/journal.pone.0047298 PMC3470570

[bib36] Guardavaccaro D Kudo Y Boulaire J Barchi M Busino L Donzelli M Control of meiotic and mitotic progression by the F box protein beta-Trcp1 *in vivo* Dev Cell 2003 4 799 812 12791266 10.1016/s1534-5807(03)00154-0

[bib37] Margottin-Goguet F Hsu JY Loktev A Hsieh HM Reimann JD Jackson PK Prophase destruction of Emi1 by the SCF(betaTrCP/Slimb) ubiquitin ligase activates the anaphase promoting complex to allow progression beyond prometaphase Dev Cell 2003 4 813 826 12791267 10.1016/s1534-5807(03)00153-9

[bib38] Bashir T Dorrello NV Amador V Guardavaccaro D Pagano M Control of the SCF(Skp2-Cks1) ubiquitin ligase by the APC/C(Cdh1) ubiquitin ligase Nature 2004 428 190 193 15014502 10.1038/nature02330

[bib39] Wei W Ayad NG Wan Y Zhang GJ Kirschner MW Kaelin WG Jr. Degradation of the SCF component Skp2 in cell-cycle phase G1 by the anaphase-promoting complex Nature 2004 428 194 198 15014503 10.1038/nature02381

[bib40] Woo SR Byun JG Kim YH Park ER Joo HY Yun M SIRT1 suppresses cellular accumulation of beta-TrCP E3 ligase via protein degradation Biochem Biophys Res Commun 2013 441 831 837 24211209 10.1016/j.bbrc.2013.10.146

[bib41] Lee J Zhou P Cullins and cancer Genes Cancer 2010 1 690 699 21127736 10.1177/1947601910382899 PMC2994581

[bib42] Zhong J Shaik S Wan L Tron AE Wang Z Sun L SCF beta-TRCP targets MTSS1 for ubiquitination-mediated destruction to regulate cancer cell proliferation and migration Oncotarget 2013 4 2339 2353 24318128 10.18632/oncotarget.1446 PMC3926831

[bib43] Duan S Skaar JR Kuchay S Toschi A Kanarek N Ben-Neriah Y mTOR generates an auto-amplification loop by triggering the betaTrCP- and CK1alpha-dependent degradation of DEPTOR Mol Cell 2011 44 317 324 22017877 10.1016/j.molcel.2011.09.005 PMC3212871

[bib44] Gao D Inuzuka H Tan MK Fukushima H Locasale JW Liu P mTOR drives its own activation via SCF(betaTrCP)-dependent degradation of the mTOR inhibitor DEPTOR Mol Cell 2011 44 290 303 22017875 10.1016/j.molcel.2011.08.030 PMC3229299

[bib45] Zhao Y Xiong X Sun Y DEPTOR, an mTOR inhibitor, is a physiological substrate of SCF(betaTrCP) E3 ubiquitin ligase and regulates survival and autophagy Mol Cell 2011 44 304 316 22017876 10.1016/j.molcel.2011.08.029 PMC3216641

